# Supplementary material for: In Vitro synergy of Farnesyltransferase inhibitors in combination with colistin against ESKAPE bacteria
Source: PLoS One. 2025 Sep 5;20(9):e0331440. doi: 10.1371/journal.pone.0331440 (PMC12412981; doi:10.1371/journal.pone.0331440)
Supplement: S1 File — The file provides supporting information on the Tecan device and the program used for fluorescence measurements, as well as details of the conducted controls. (DOCX) [file pone.0331440.s001.docx]

First, additional information on the Tecan device is given, as the program used for the evaluation is shown. The controls conducted are then presented. In addition to the antibiotics ampicillin, chloramphenicol, kanamycin and tetracycline, colistin was also tested. Relevant concentrations of DMSO and ethanol were additionally included in the test. It can therefore be ruled out that these substances, in which the active ingredients were partially dissolved, had an influence on the results. The pipetting scheme is shown in advance. Bacterial strains, abbreviations, and chemicals follow the definitions provided in the main publication.


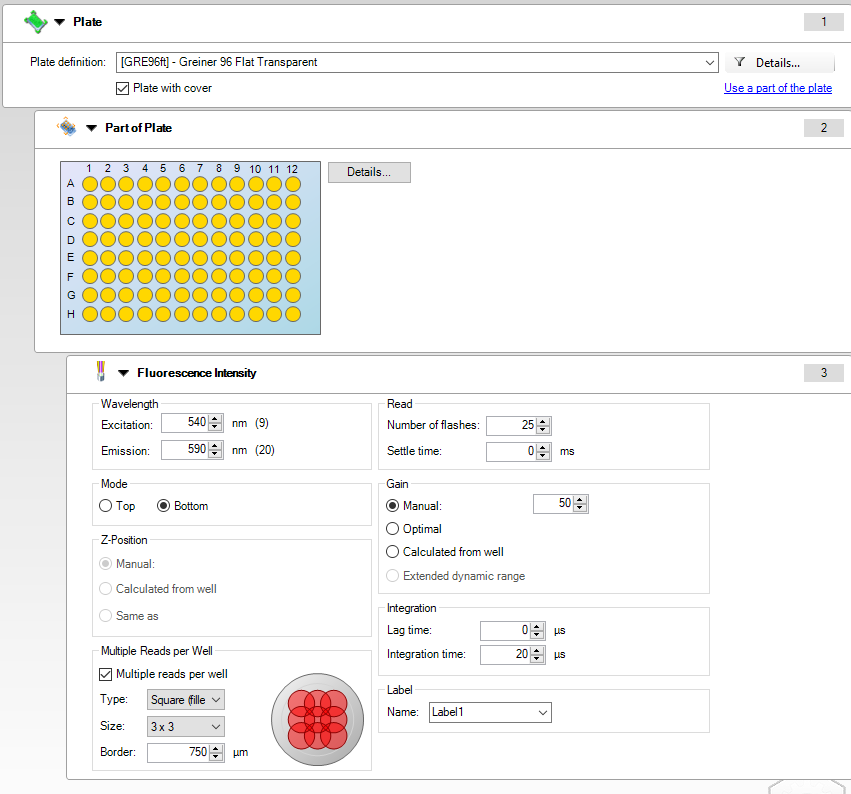


**Program overview for the Tecan microplate reader (i-control 2.0 software).**

Multiple reads per well are recommended. Gain was set to 50 for all runs after testing different settings to optimize sensitivity and prevent signal saturation. The relative fluorescence intensity (RFI) for triplicate samples can then be evaluated using the following formula:

$$RFI= \left( \frac{\left( \frac{X_{n1}+X_{n2}+X_{n3}}{3} \right)-X_{NC}}{\left( \frac{{PC}_{n1}+{PC}_{n2}+{PC}_{n3}}{3} \right)-X_{NC}} \right)*100$$

$X$ represents the fluorescence intensity of a specific concentration or drug combination, with $X_{n1}$, $X_{n2}$, $X_{n3}$ denoting the triplicate technical measurements. The negative control (NC) accounts for background fluorescence, while the positive control (PC) represents maximum bacterial growth in the absence of antimicrobial agents. The calculated RFI values are expressed as a percentage, where the positive control is set to 100.0, and all other results are relative to this reference.


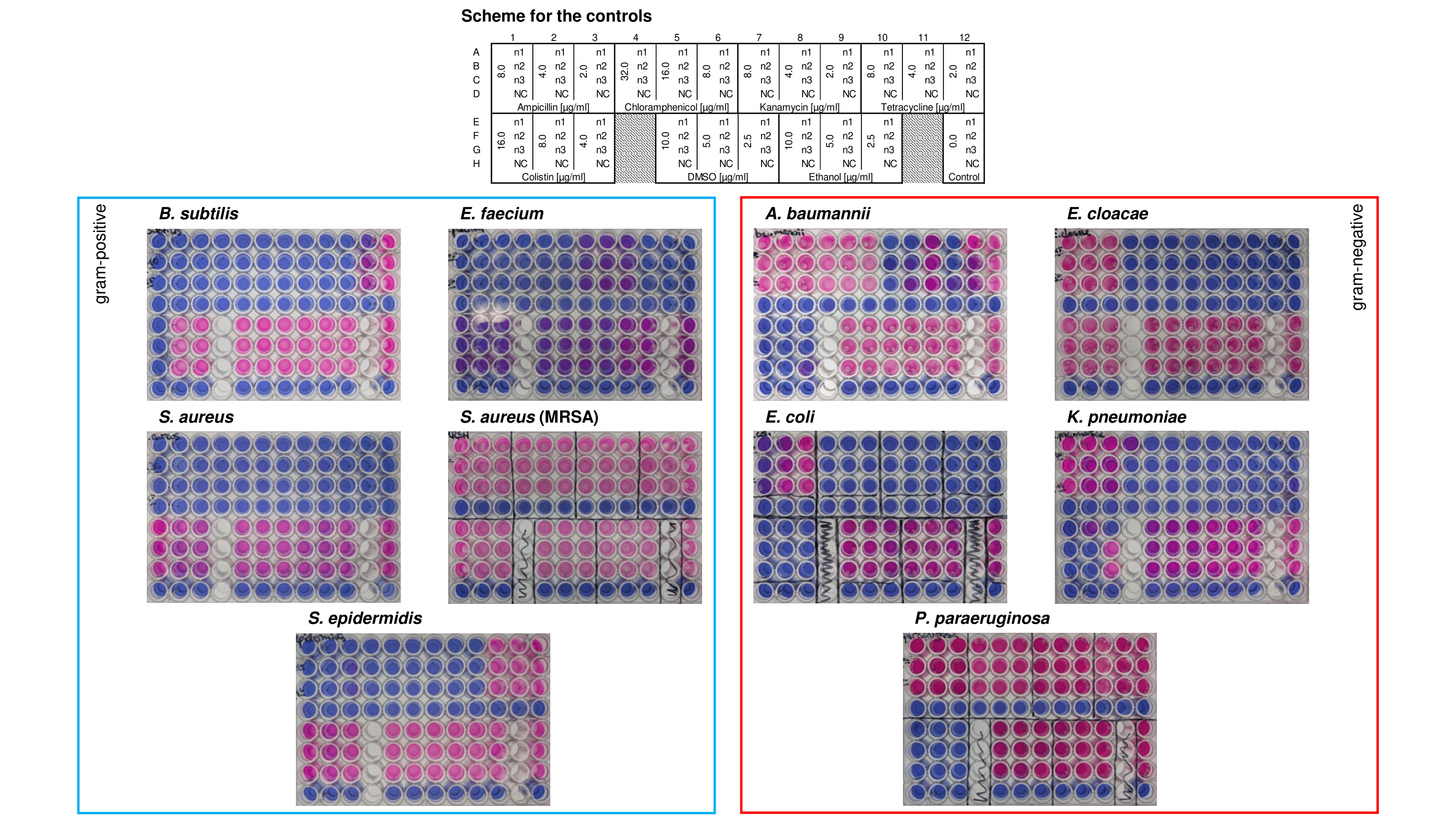


**Control setup and pipetting scheme for the tested bacterial strains.**
