# Supplementary figures and images for: In Vitro synergy of Farnesyltransferase inhibitors in combination with colistin against ESKAPE bacteria
Source: PLoS One. 2025 Sep 5;20(9):e0331440. doi: 10.1371/journal.pone.0331440 (PMC12412981; doi:10.1371/journal.pone.0331440)

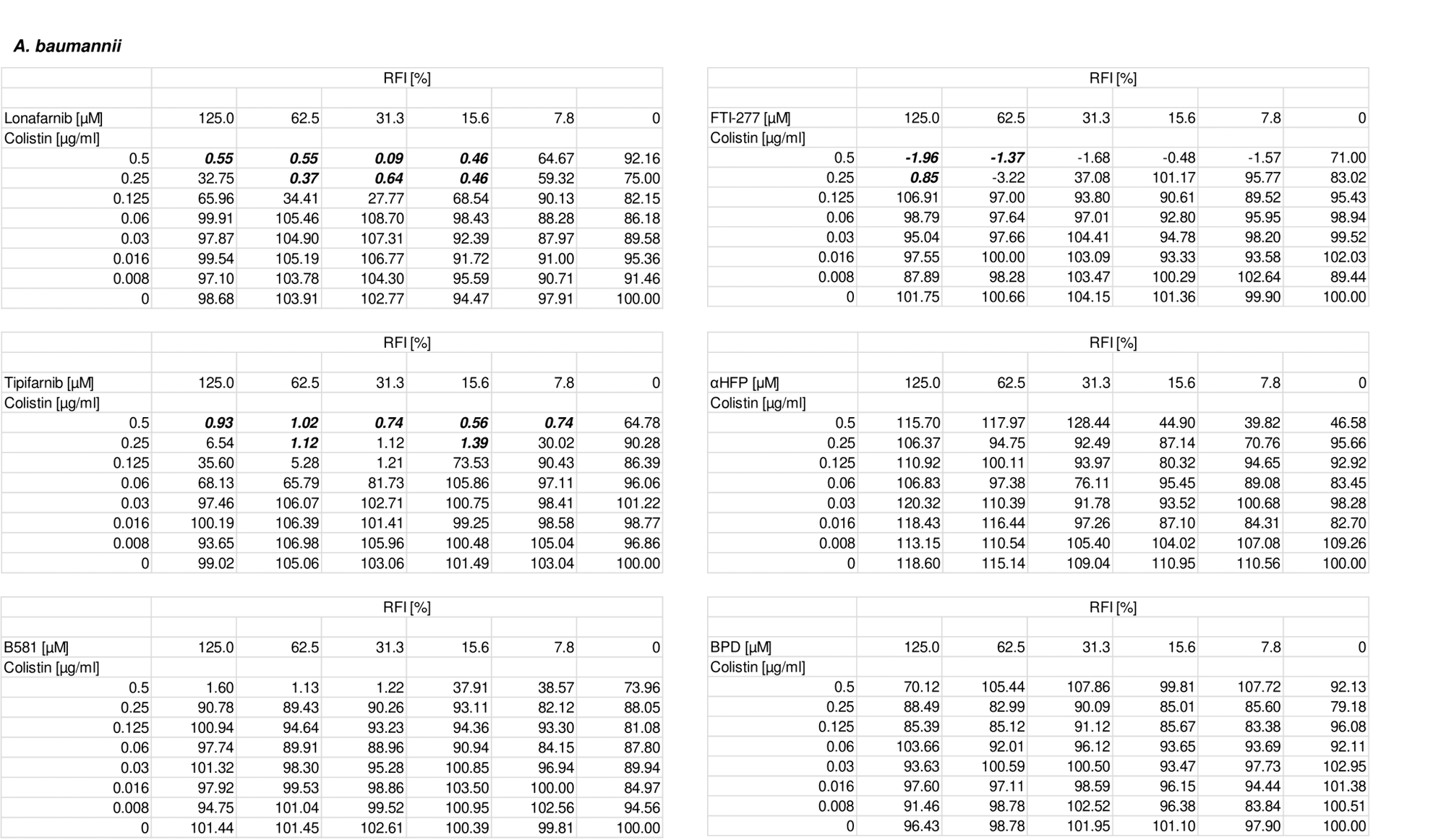

Supplement: S1 Fig — Relative fluorescence intensity (RFI) values for all checkerboard assays with colistin and the six tested substances for A. baumannii. Minimum bactericidal concentrations (MBCs) are indicated in bold and italic. All values were normalized to the fluorescence of the corresponding growth control to ensure comparability across plates. All checkerboard assays were conducted in triplicate. (TIF) [file pone.0331440.s002.tif]

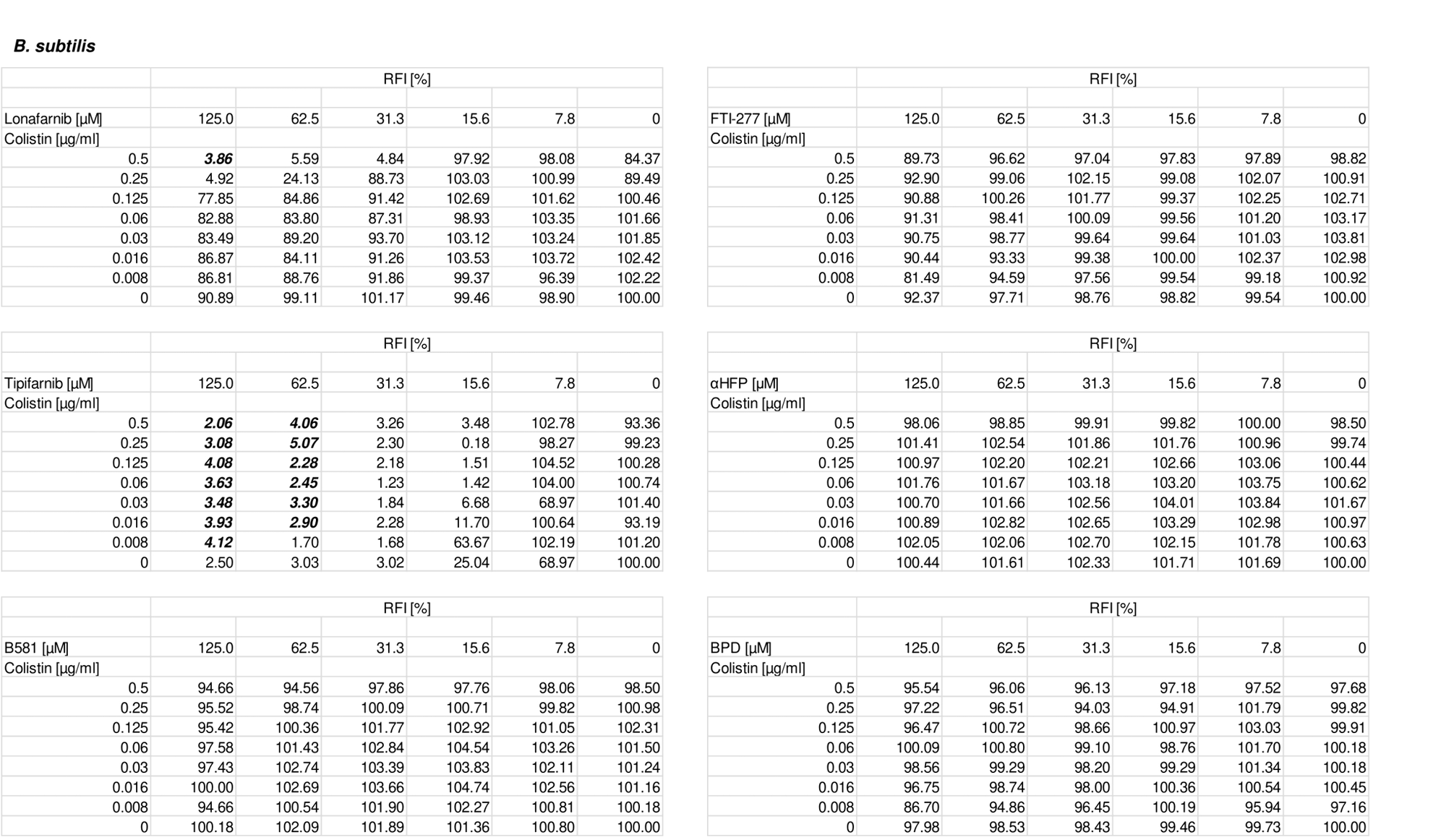

Supplement: S2 Fig — Relative fluorescence intensity (RFI) values for all checkerboard assays with colistin and the six tested substances for B. subtilis. Minimum bactericidal concentrations (MBCs) are indicated in bold and italic. All values were normalized to the fluorescence of the corresponding growth control to ensure comparability across plates. All checkerboard assays were conducted in triplicate. (TIF) [file pone.0331440.s003.tif]

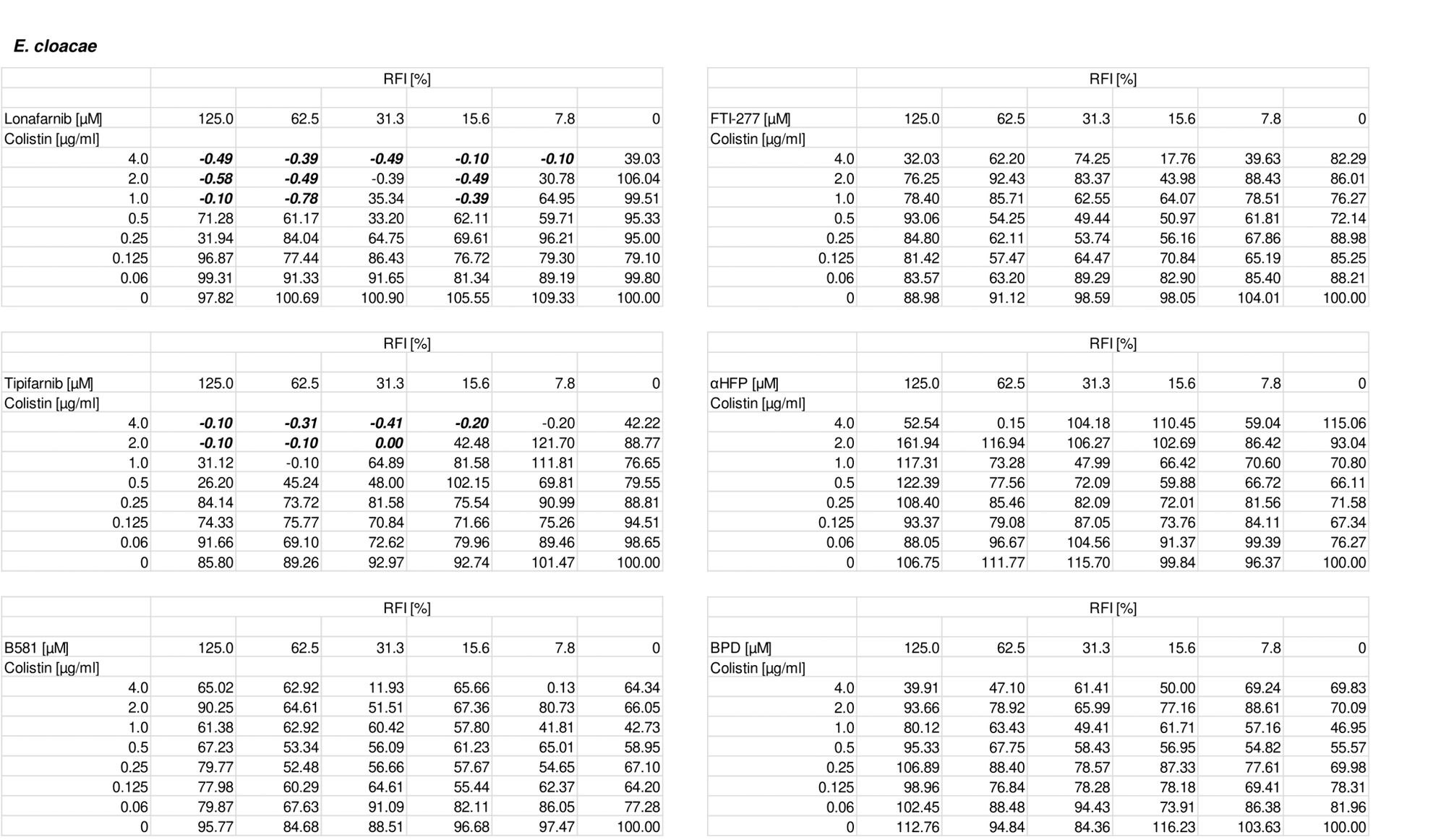

Supplement: S3 Fig — Relative fluorescence intensity (RFI) values for all checkerboard assays with colistin and the six tested substances for E. cloacae. Minimum bactericidal concentrations (MBCs) are indicated in bold and italic. All values were normalized to the fluorescence of the corresponding growth control to ensure comparability across plates. All checkerboard assays were conducted in triplicate. (TIF) [file pone.0331440.s004.tif]

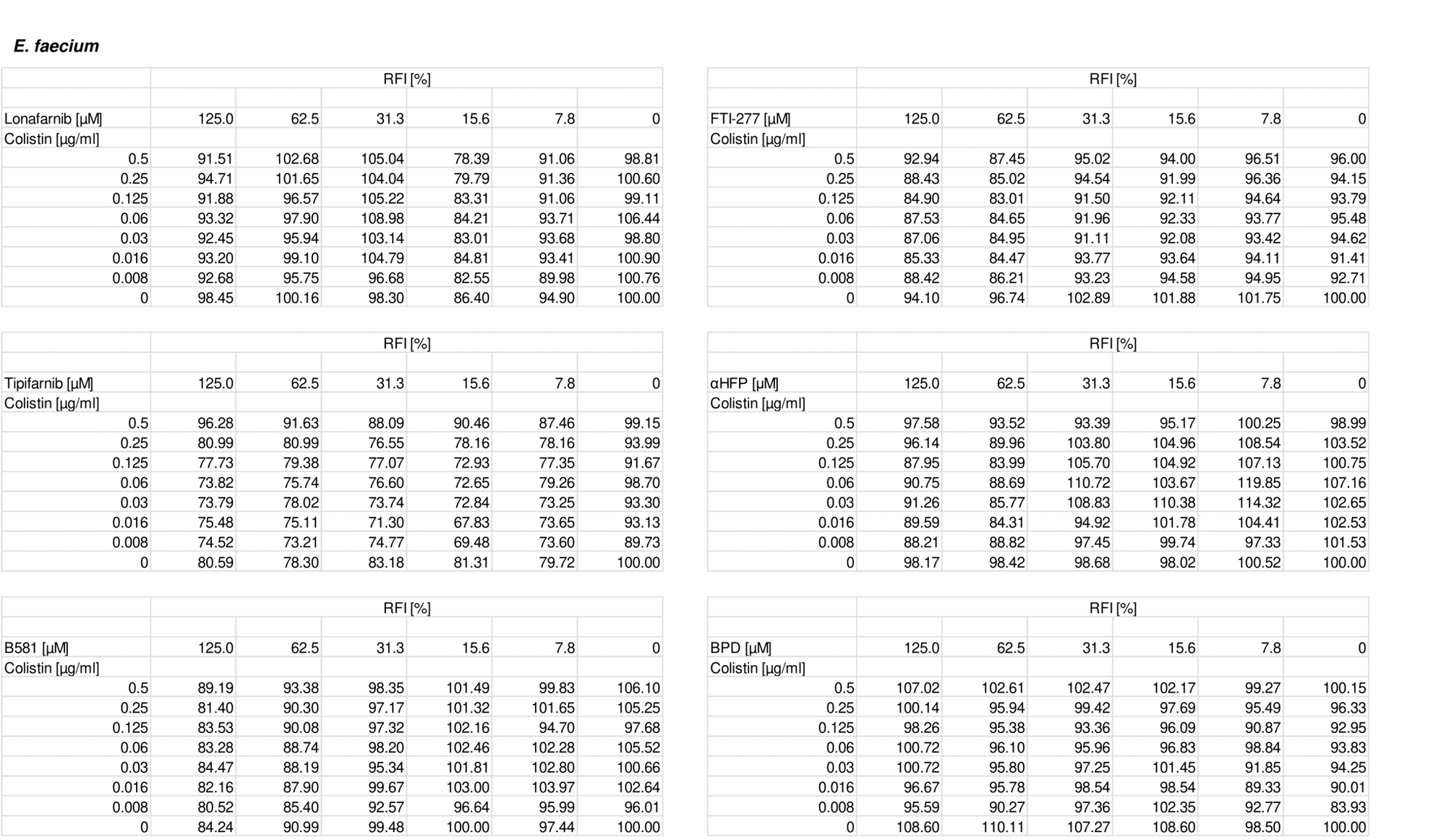

Supplement: S4 Fig — Relative fluorescence intensity (RFI) values for all checkerboard assays with colistin and the six tested substances for E. faecium Minimum bactericidal concentrations (MBCs) are indicated in bold and italic. All values were normalized to the fluorescence of the corresponding growth control to ensure comparability across plates. All checkerboard assays were conducted in triplicate. (TIF) [file pone.0331440.s005.tif]

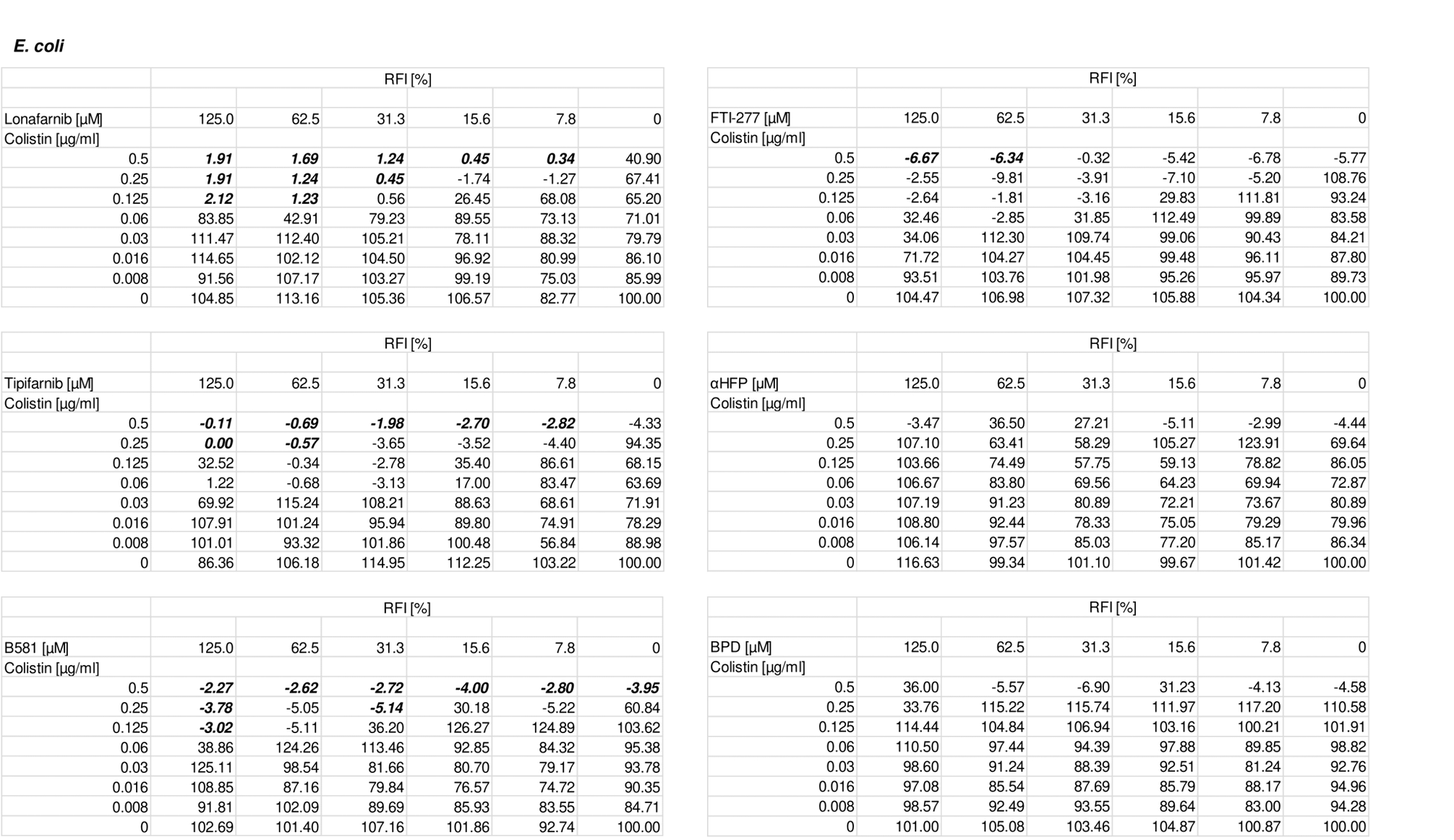

Supplement: S5 Fig — Relative fluorescence intensity (RFI) values for all checkerboard assays with colistin and the six tested substances for E. coli. Minimum bactericidal concentrations (MBCs) are indicated in bold and italic. All values were normalized to the fluorescence of the corresponding growth control to ensure comparability across plates. All checkerboard assays were conducted in triplicate. (TIF) [file pone.0331440.s006.tif]

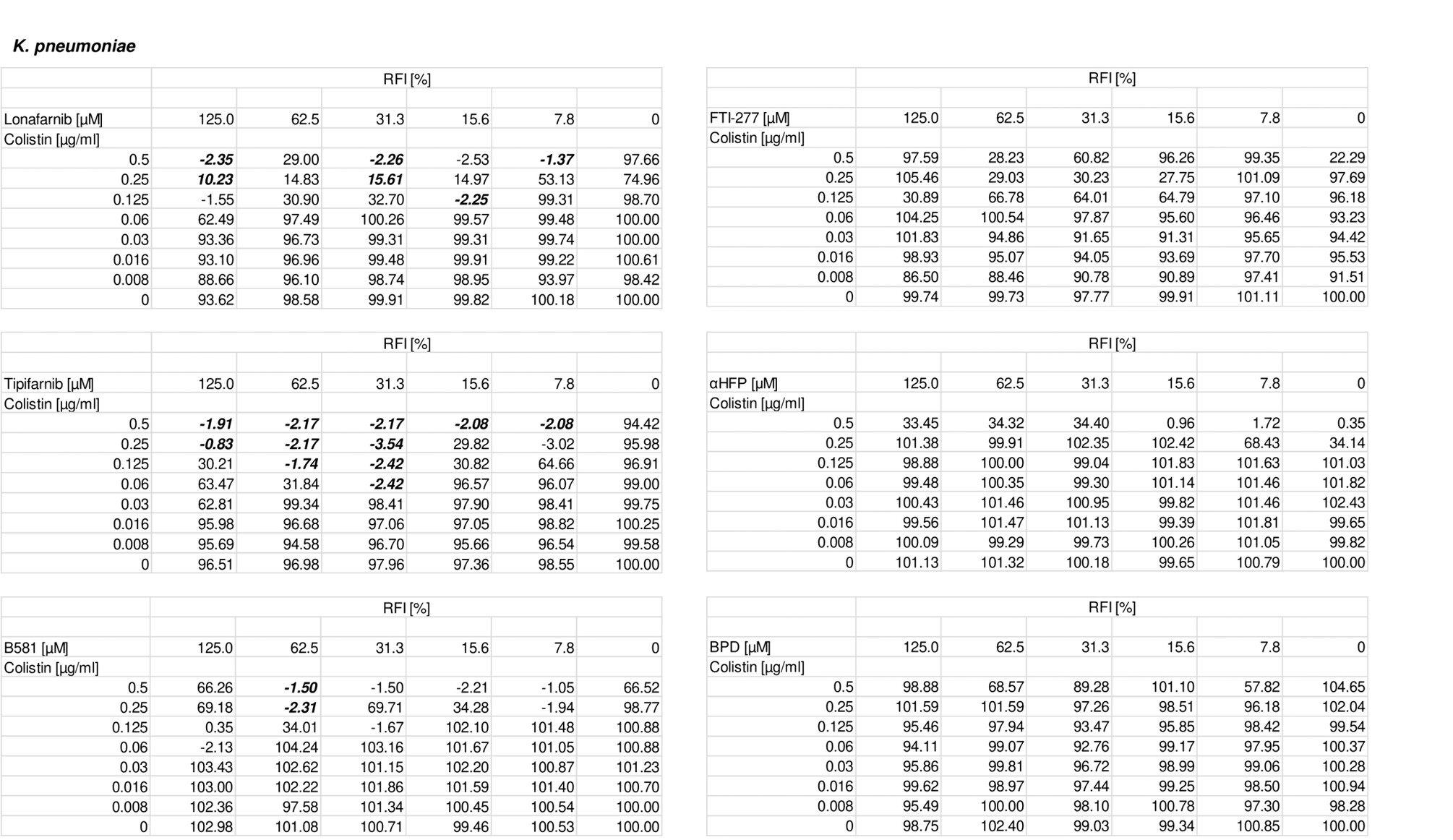

Supplement: S6 Fig — Relative fluorescence intensity (RFI) values for all checkerboard assays with colistin and the six tested substances for K. pneumoniae. Minimum bactericidal concentrations (MBCs) are indicated in bold and italic. All values were normalized to the fluorescence of the corresponding growth control to ensure comparability across plates. All checkerboard assays were conducted in triplicate. (TIF) [file pone.0331440.s007.tif]

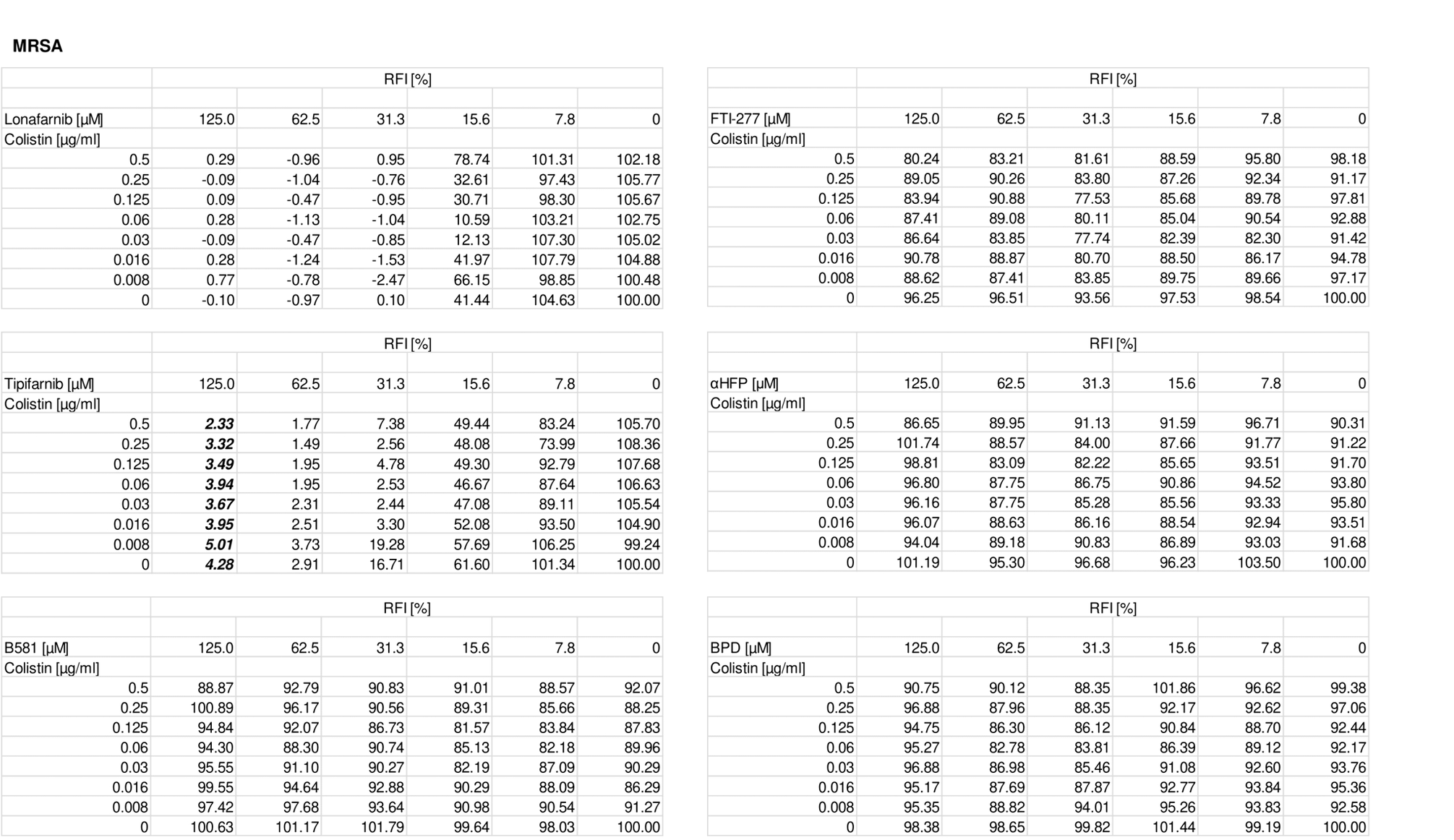

Supplement: S7 Fig — Relative fluorescence intensity (RFI) values for all checkerboard assays with colistin and the six tested substances for MRSA. Minimum bactericidal concentrations (MBCs) are indicated in bold and italic. All values were normalized to the fluorescence of the corresponding growth control to ensure comparability across plates. All checkerboard assays were conducted in triplicate. (TIF) [file pone.0331440.s008.tif]

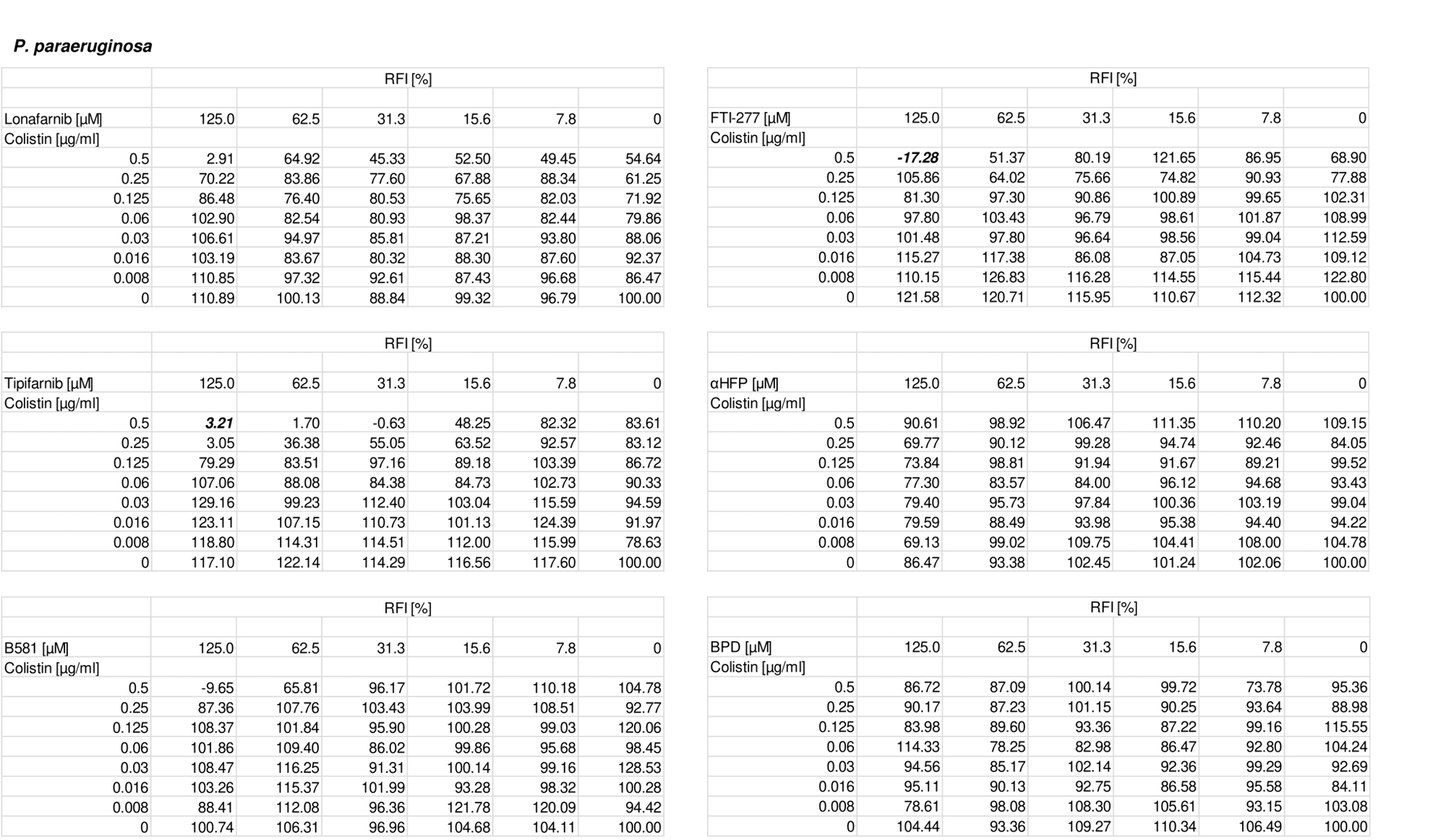

Supplement: S8 Fig — Relative fluorescence intensity (RFI) values for all checkerboard assays with colistin and the six tested substances for P. paraeruginosa. Minimum bactericidal concentrations (MBCs) are indicated in bold and italic. All values were normalized to the fluorescence of the corresponding growth control to ensure comparability across plates. All checkerboard assays were conducted in triplicate. (TIF) [file pone.0331440.s009.tif]

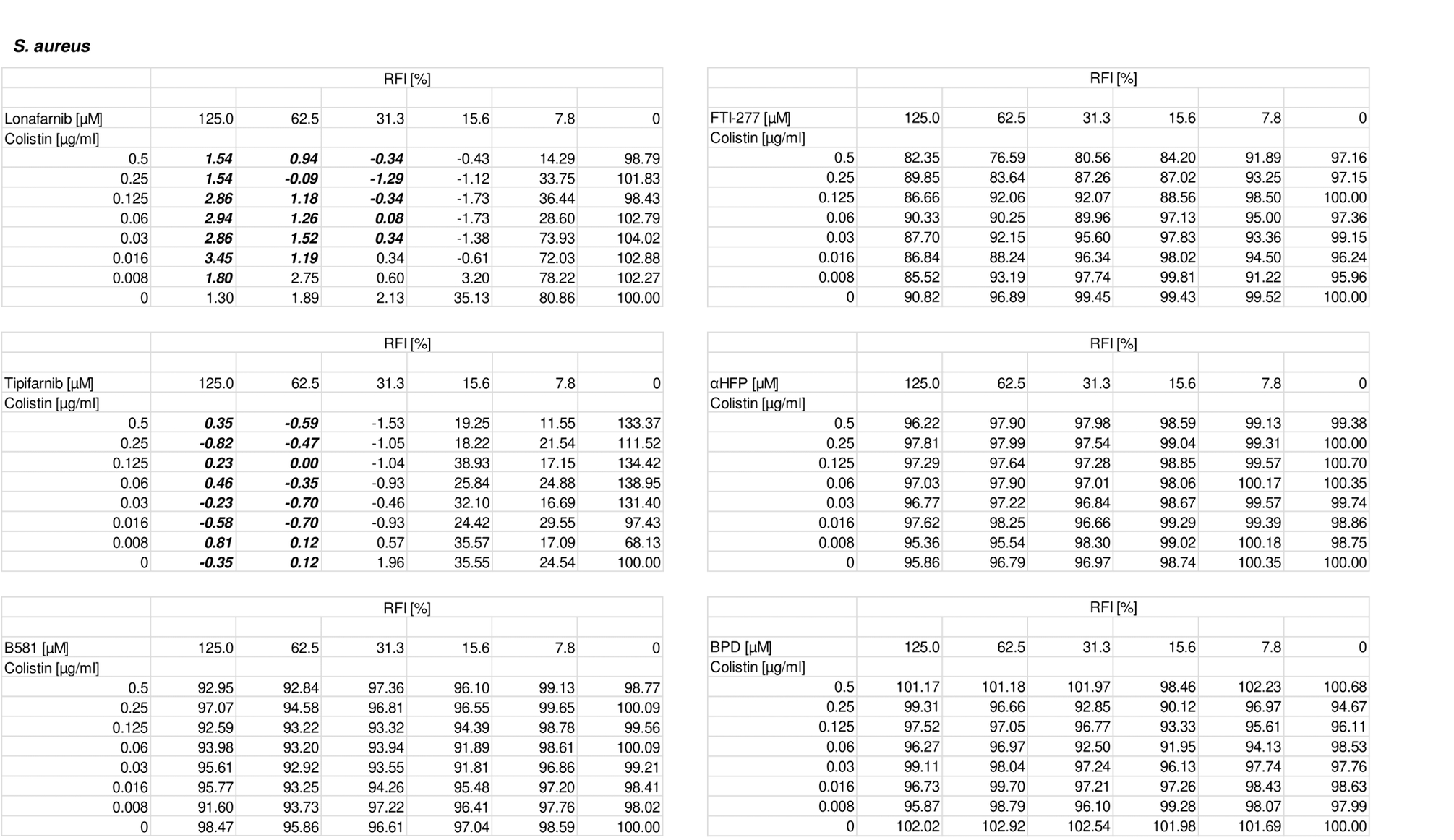

Supplement: S9 Fig — Relative fluorescence intensity (RFI) values for all checkerboard assays with colistin and the six tested substances for S. aureus. Minimum bactericidal concentrations (MBCs) are indicated in bold and italic. All values were normalized to the fluorescence of the corresponding growth control to ensure comparability across plates. All checkerboard assays were conducted in triplicate. (TIF) [file pone.0331440.s010.tif]

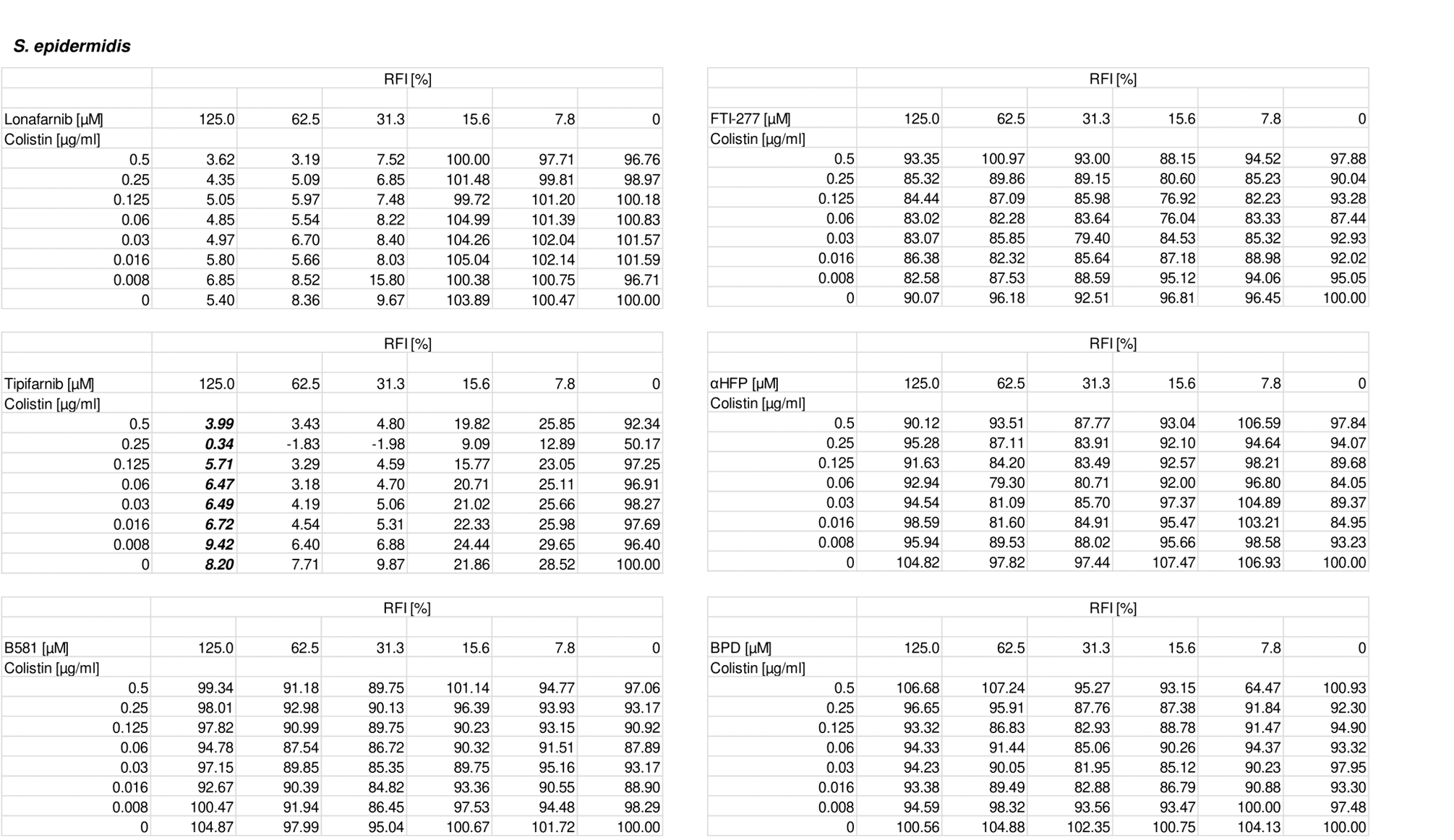

Supplement: S10 Fig — Relative fluorescence intensity (RFI) values for all checkerboard assays with colistin and the six tested substances for S. epidermidis. Minimum bactericidal concentrations (MBCs) are indicated in bold and italic. All values were normalized to the fluorescence of the corresponding growth control to ensure comparability across plates. All checkerboard assays were conducted in triplicate. (TIF) [file pone.0331440.s011.tif]
